# Cardiovascular Disease, Atherosclerosis and Familial Hypercholesterolemia: From Molecular Mechanisms Causing Pathogenicity to New Therapeutic Approaches

**DOI:** 10.3390/ijms24087659

**Published:** 2023-04-21

**Authors:** Shifa Jebari-Benslaiman, Asier Larrea-Sebal, Asier Benito-Vicente, César Martín

**Affiliations:** 1 Department of Biochemistry and Molecular Biology, Universidad del País Vasco UPV/EHU, 48080 Bilbao, Spain; shifa.jebari@ehu.eus (S.J.-B.); asierlarrea@yahoo.es (A.L.-S.); asier.benito@ehu.eus (A.B.-V.); 2 Department of Molecular Biophysics, Biofisika Institute, University of Basque Country and Consejo Superior de Investigaciones Científicas (UPV/EHU, CSIC), 48940 Leioa, Spain; 3 Fundación Biofisika Bizkaia, 48940 Leioa, Spain

This Special Issue, “Cardiovascular Disease, Atherosclerosis and Familial Hypercholesterolemia: From Molecular Mechanisms Causing Pathogenicity to New Therapeutic Approaches”, contributes to advancing our knowledge of the molecular mechanisms that drive cardiovascular disease, atherosclerosis and familial hypercholesterolemia and the development of state-of-the-art research in the field. This Special Issues is the result of a call for papers that was sent out in late 2020 and continued till mid-2022, from which twelve papers (six original research articles, one hypothesis and five reviews) were published.

In this context, Unai Galicia-Garcia et al. provide a comprehensive overview of the pathophysiology of type 2 diabetes mellitus (T2D) and its relationship with cardiovascular disease. The authors present a detailed analysis of the mechanisms linking insulin resistance and inflammation to the development of atherosclerosis, a major risk factor for cardiovascular disease. The paper also discusses the potential therapeutic approaches for managing T2D and reducing the risk of cardiovascular complications. The review also examines the molecular mechanisms that contribute to insulin resistance, which include the disruption of the insulin signaling pathway, inflammation and oxidative stress. Additionally, the paper investigates the influence of both genetic and environmental factors in the pathogenesis of T2D [1]. This work is further complemented by a review that explores the potential mechanisms by which statins may contribute to T2D development. Galicia-Garcia et al. [2] discuss clinical evidence linking statin use to an increased risk of T2D and present several possible mechanisms, including alterations in insulin signaling, glucose metabolism and mitochondrial function. The review also considers the role of genetic susceptibility and environmental factors in the development of statin-induced T2D [2]. Overall, the two reviews complement each other by highlighting the complex interplay between genetic and environmental factors in the development of T2D and the importance of personalized approaches to treatment and prevention [1,2].

The potential secondary effects derived from statin treatment are further explored in this Special Issue by the hypothesis work of Ruiz-Pesini et al. [3]. This study provides valuable insights into the potential role of a mitochondrial genetic background in the development of atherosclerosis and statin-induced side effects in patients with familial hypercholesterolemia (FH). Ruiz-Pesini et al. highlight the fact that FH can be caused by various genetic mutations, and the variability in disease severity and response to statin therapy suggests the involvement of other factors that modulate disease development. The authors suggest that mitochondrial DNA variants and copy numbers may serve as potential markers for predicting cardiovascular disease development and statin-induced side effects in FH patients. In addition, they discuss the potential role of CoQ10 in atherosclerosis development and present several genes that could affect its regulation and bioavailability. This research proposes a personalized medicine approach to FH therapy, which could have important clinical implications for the management of this disease.

Challenges in diagnosing coronary artery disease (CAD) in the elderly population are reviewed by Kozlov et al. in the paper “Noninvasive Testing for Diagnosis of Stable Coronary Artery Disease in the Elderly” [4]. This age group is vulnerable due to its high prevalence of comorbidities, contraindications to exercise tests and cognitive decline, which can make assessing their condition difficult. The authors discuss the current recommendations of the US and European cardiologists’ associations and their applicability for diagnostics in the elderly population. The authors highlight that exercise electrocardiogram (ECG) and exercise stress echocardiography (SE) tests are not feasible for a substantial proportion of elderly patients. Coronary computed tomography angiography (CTA) seems to be a promising option for these patients, but its suitability is not universal. It may present difficulties in cases where there is notable vessel calcification. Additionally, further research is necessary to compare CTA outcomes with those of other diagnostic approaches. It is emphasized that future research should focus on improving diagnostic criteria and conducting comparative studies to better understand the benefits and drawbacks of different diagnostic approaches.

Additionally, within the context of the pathogenesis of cardiovascular diseases (CVDs), Blanda et al. review the role of galectin-3 (Gal-3), a β-galactoside-binding protein belonging to the lectin family with several physiological functions, such as cellular growth, proliferation, apoptosis, differentiation, cellular adhesion and tissue repair [5]. The authors provide an overview of the evidence linking Gal-3 to CVDs such as atherosclerosis, heart failure and atrial fibrillation. They describe the pro-inflammatory role of Gal-3 in the atherogenic process, its association with plaque features linked to lesion stability and its involvement in the development of cardiac fibrosis and impaired myocardium remodeling. The potential diagnostic and prognostic applications of Gal-3 in CVDs and the possibility of using Gal-3 inhibition as a therapeutic approach to preventing cardiac inflammation and fibrosis is also explored. Overall, this review article provides valuable insights into the role of Gal-3 in the pathogenesis of CVDs and highlights the potential clinical applications of Gal-3 as a diagnostic and prognostic marker and as a therapeutic target for CVDs.

Jebari-Bensaliman et al. [6] provide an in-depth review of the different stages of atherosclerosis development, ranging from endothelial dysfunction to plaque rupture. The review explores the role of endothelial activation and the cascade of events that follow, including the accumulation of lipids, fibrous elements and calcification, which triggers the narrowing of vessels and inflammatory pathways and emphasizes the importance of identifying and managing risk factors, including sex, which is a crucial risk factor in atherosclerosis. Moreover, the review covers the post-transcriptional regulation and modulation of atheroma plaque by microRNAs and lncRNAs and the role of the microbiota in atherosclerosis. It discusses potential therapeutic interventions and the importance of continued research to better understand the pathophysiology of atherosclerosis and develop effective treatments to prevent and manage this disease.

Regarding research articles, this Special Issue has published a number of compelling articles that hold great relevance for the field of cardiovascular disease.

The research paper by Maeng et al. explores the role of fibroblast growth factor 21 (FGF21) in atherosclerosis development and associated lipid metabolic profiles [7]. The study was conducted using ApoE^−/−^ mice, which were fed an atherogenic diet and treated with an FGF21 analogue (LY2405319), and provides valuable insights into the potential role of FGF21 in atherosclerosis development and its associated lipid metabolic profiles. The results showed that treatment with LY2405319 reduced atherosclerotic plaque formation and improved lipid metabolic profiles, including decreasing plasma triglycerides. FGF21 administration was found to have a positive impact on key pathways related to atherosclerosis and exerted anti-atherosclerotic effects in mice with a higher risk of developing the condition. It seems that an improvement in inflammation and insulin resistance are among the mechanisms responsible for the beneficial role of FGF21 therapy in reducing atherosclerosis. These findings suggest that FGF21 could be a potential therapeutic target for preventing atherosclerosis.

Continuing with the exploration of biomolecules with potential therapeutic use in preventing atherosclerosis, Ku et al. investigated the effect of anakinra, a recombinant human interleukin-1 receptor antagonist, on the progression of atherosclerosis in ApoE^−/−^ mice [8]. Their results show that anakinra reduced the plaque size of the aortic arch and serum triglyceride levels in ApoE^−/−^ mice. It also suppressed the expression of inflammatory genes (IL-1β and IL-6) in HUVECs and RAOSMCs. In RAOSMCs, anakinra reduced metalloproteinase-9 expression in a dose-dependent manner and inhibited cell migration. Anakinra-treated mice also exhibited trends of lower CD68+ macrophage infiltration in visceral fat, and monocyte chemoattractant protein-1 expression was reduced in 3T3-L1 adipocytes. According to the findings, incorporating anakinra into a standard treatment regimen could be beneficial in reducing the remaining cardiovascular risk.

The relationship between non-fasting triglyceride (TG) levels and the progression of carotid artery stenosis in patients with atherosclerosis was investigated by Miura et al. [9]. The study showed that a new parameter, the area [TG ≥ 175], which integrates non-fasting TG values with the measurement intervals, is a significant factor in predicting stenosis progression. Miura et al. also found that controlling non-fasting TG levels below 175 mg/dL is crucial to prevent carotid stenosis progression [9], highlighting the importance of monitoring TG levels in patients with atherosclerosis to prevent cardiovascular disease progression. Overall, this study provides valuable insights into residual cardiovascular risk factors and potential strategies for preventing atherosclerosis progression.

The discovery of biomarkers is crucial for the diagnosis and prevention of cardiovascular disease as they can provide valuable information about disease risk, severity and progression. Dziedzic et al. investigated the relationship between novel inflammatory biomarkers, the Systemic Inflammatory Index (SII) and the Systemic Inflammatory Response Index (SIRI) and the severity of coronary artery disease (CAD) as well as the occurrence of acute coronary syndrome (ACS) [10]. The obtained results indicate that the SIRI, but not the SII, is significantly associated with the diagnosis of ACS, with the highest values observed in patients with ST-segment elevation myocardial infarction (STEMI). Additionally, the highest SII and SIRI values were observed in patients with three-vessel CAD. These findings suggest that the SII and SIRI may have potential as clinical biomarkers for predicting the severity of CAD and ACS occurrence.

In their study, Matloch et al. [11] investigated the potential association between decreased epicardial CTRP3 mRNA levels and the presence of coronary artery disease (CAD) in patients with type 2 diabetes mellitus (T2DM) undergoing elective cardiac surgery. C1q TNF-related protein 3 (CTRP3) is an adipokine that has been shown to have anti-inflammatory and cardioprotective properties. The authors assessed CTRP3 mRNA levels in epicardial and subcutaneous adipose tissue, as well as circulating levels of CTRP3 and other factors in three groups of patients with different disease profiles. The findings suggest that baseline CTRP3 mRNA levels in epicardial adipose tissue were significantly decreased in patients with both CAD and T2DM, but not in those with CAD alone or without either disease. This result indicates that decreased epicardial CTRP3 mRNA levels may be associated with a higher risk of atherosclerosis in patients with both CAD and T2DM.

Finally, Larrea-Sebal et al. [12] conducted a functional characterization of a genetic variant in the *Protein convertase subtilisin/kexin type 9* (*PCSK9*) gene, which was detected in a patient with suspected familial hypercholesterolemia (FH). Various techniques, including autocatalytic cleavage efficiency, protein expression and an LDLr activity assay, were employed to assess the functionality of the PCSK9 variant. The results demonstrate that the p.(Arg160Gln) PCSK9 variant is a loss-of-function (LOF) variant that reduces the stability of the LDLr–PCSK9 complex. This study highlights the significance of functionally characterizing genetic variants to aid in the genetic diagnosis of FH.

The authors’ contributions, whether through original experimental studies, clinical studies or literature reviews, are essential to making this Special Issue a valuable resource for researchers interested in exploring molecular mechanisms associated with cardiovascular disorders, as well as in developing personalized therapeutic strategies and improving diagnostic methods.

## References

[1] Galicia-Garcia U., Benito-Vicente A., Jebari S., Larrea-Sebal A., Siddiqi H., Uribe K.B., Ostolaza H., Martin C. (2020). Pathophysiology of Type 2 Diabetes Mellitus. Int. J. Mol. Sci..

[2] Galicia-Garcia U., Jebari S., Larrea-Sebal A., Uribe K.B., Siddiqi H., Ostolaza H., Benito-Vicente A., Martin C. (2020). Statin Treatment-Induced Development of Type 2 Diabetes: From Clinical Evidence to Mechanistic Insights. Int. J. Mol. Sci..

[3] Ruiz-Pesini E., Bayona-Bafaluy M.P., Sanclemente T., Puzo J., Montoya J., Pacheu-Grau D. (2022). Mitochondrial Genetic Background May Impact Statins Side Effects and Atherosclerosis Development in Familial Hypercholesterolemia. Int. J. Mol. Sci..

[4] Kozlov S.G., Chernova O.V., Gerasimova E.V., Ivanova E.A., Orekhov A.N. (2020). Noninvasive Testing for Diagnosis of Stable Coronary Artery Disease in the Elderly. Int. J. Mol. Sci..

[5] Blanda V., Bracale U.M., Di Taranto M.D., Fortunato G. (2020). Galectin-3 in Cardiovascular Diseases. Int. J. Mol. Sci..

[6] Jebari-Benslaiman S., Galicia-Garcia U., Larrea-Sebal A., Olaetxea J.R., Alloza I., Vandenbroeck K., Benito-Vicente A., Martin C. (2022). Pathophysiology of Atherosclerosis. Int. J. Mol. Sci..

[7] Maeng H.J., Lee G.Y., Bae J.H., Lim S. (2020). Effect of Fibroblast Growth Factor 21 on the Development of Atheromatous Plaque and Lipid Metabolic Profiles in an Atherosclerosis-Prone Mouse Model. Int. J. Mol. Sci..

[8] Ku E.J., Kim B.R., Lee J.I., Lee Y.K., Oh T.J., Jang H.C., Choi S.H. (2022). The Anti-Atherosclerosis Effect of Anakinra, a Recombinant Human Interleukin-1 Receptor Antagonist, in Apolipoprotein E Knockout Mice. Int. J. Mol. Sci..

[9] Miura Y., Yasuda R., Toma N., Suzuki H. (2022). Non-Fasting Hypertriglyceridemia Burden as a Residual Risk of the Progression of Carotid Artery Stenosis. Int. J. Mol. Sci..

[10] Dziedzic E.A., Gasior J.S., Tuzimek A., Paleczny J., Junka A., Dabrowski M., Jankowski P. (2022). Investigation of the Associations of Novel Inflammatory Biomarkers-Systemic Inflammatory Index (SII) and Systemic Inflammatory Response Index (SIRI)-With the Severity of Coronary Artery Disease and Acute Coronary Syndrome Occurrence. Int. J. Mol. Sci..

[11] Matloch Z., Mraz M., Kasperova B.J., Kratochvilova H., Svoboda P., Pleyerova I., Reznickova K., Norman S., Hlavacek D., Mahrik J. (2022). Decreased Epicardial CTRP3 mRNA Levels in Patients with Type 2 Diabetes Mellitus and Coronary Artery Disease Undergoing Elective Cardiac Surgery: A Possible Association with Coronary Atherosclerosis. Int. J. Mol. Sci..

[12] Larrea-Sebal A., Trenti C., Jebari-Benslaiman S., Bertolini S., Calandra S., Negri E.A., Bonelli E., Benito-Vicente A., Uraga-Gracianteparaluceta L., Martin C. (2023). Functional Characterization of p.(Arg160Gln) PCSK9 Variant Accidentally Found in a Hypercholesterolemic Subject. Int. J. Mol. Sci..

